# Expectation of Selfishness From Others in Borderline Personality Disorder

**DOI:** 10.3389/fpsyg.2021.702227

**Published:** 2021-08-19

**Authors:** Erika Evelyn Lévay, Bettina Bajzát, Zsolt Szabolcs Unoka

**Affiliations:** Department of Psychiatry and Psychotherapy, Semmelweis University, Budapest, Hungary

**Keywords:** social value orientation, borderline personality disorder, trust, social cognition, intentions of others, mentalization

## Abstract

Social difficulties are apparent in borderline personality disorder (BPD). Behavior in BPD is characterized by mistrust and expectations of malevolence from others. We examined whether there is an asymmetry between their social behavior and their belief about other people’s social motivations. Subjects completed a task where they had to allocate money between themselves and an imagined other they will not meet and interact with. In addition they also had to report their expectations about how the imagined other would solve the task. We hypothesized that even though BPD patients will act in a prosocial way, they will expect selfish behavior from the other. We used the Slider Measure of social value orientation (SVO) and also created a modified version of the measure to examine the discrepancy between the subjects’ own SVO and their expectations from other people. We compared the results of thirty clinically diagnosed BPD patients to a matched sample of healthy participants. Our results show that the BPD group’s selfishness expectations significantly outweigh the expectations of selfishness in the HC group (*U* = 269, *p* = 0.007). This result further supports the mistrust and negativity bias observed in various aspects of social interactions in BPD.

## Introduction

Patients with borderline personality disorder (BPD) show intense reactions to perceived abandonment, a high degree of mistrust, and a distorted, negative perception of others (American Psychiatric Association, 2013). Difficulties in mentalization, misunderstanding others’ mental state, especially in the state of emotional arousal and in the context of attachment, is a crucial problem in BPD (Fonagy and Bateman, 2008) that contribute significantly to interpersonal problems (Euler et al., 2019).

Although there are reports about enhanced or preserved emotion recognition and mentalization in BPD (e.g., Fertuck et al., 2009), increasing the complexity of mentalization tasks can highlight the difficulties of BPD patients (Minzenberg et al., 2006; Preißler et al., 2010). The majority of findings point to the direction that people with BPD do have mentalization impairments (Salgado et al., 2020). They misinterpret social cues with a pronounced negative bias (Roepke et al., 2013) or fail to accurately perceive positive and neutral cues (Unoka et al., 2014). Negative bias appears when judging traits like approachability and trustworthiness of a person from a photo (Fertuck et al., 2013; Miano et al., 2013; Nicol et al., 2013) and is present in the detection of facial expressions (Dyck et al., 2009; Unoka et al., 2011), even after remission of the disorder (Kleindienst et al., 2019). Barnow et al. (2009) found that people with BPD gave a more negative estimate of others’ character and even a more aggressive one than healthy controls and depressed participants. Findings by Giesen-Bloo and Arntz (2005) also show that patients with BPD assume the world and others significantly more malevolent than patients with other mental disorders (Cluster C and Axis I) or the healthy subjects.

The general tendency to negatively evaluate others can sabotage various forms of social interactions, and this possibly contributes to the results of previous research using economic games that shows a lack of cooperation and signs of mistrust in BPD (King-Casas et al., 2008; Seres et al., 2009; Unoka et al., 2009). In the study conducted by Unoka et al. (2009), participants were investors in 5-round trust games (TG) where they interacted with a “partner” and in 5-round risk games where their “rewards” depended on luck. BPD patients invested significantly less in the TG than healthy controls and depressed patients. However, this difference was not observable in the risk game. King-Casas et al. (2008) found that BPD subjects as trustees were unable to repair cooperation in a series of TGs and concluded that this was due to their failure to recognize the partner’s low offerings as inadequate; that is, low offerings did not fall short of their expectations about the partner’s behavior. Similar results emerged in a paper examining ultimatum game (UG) behavior in BPD patients and healthy controls: throughout the UG the BPD group refused offers at significantly lower rates. They were less influenced by the emotional expressions of the proposer than the controls (Polgár et al., 2014). Findings by Franzen et al. (2011) in a study of TG with fair and unfair virtual partners indicate that BPD patients are less inclined to modify their behavior in the social interaction depending on the partner’s emotional expression. They rely on the experience about the partner’s fairness instead.

Abramov et al. (2020) examined an undergraduate sample with high, low, and average levels of BPD traits in a 15-round trust game with participants as investors and a computer program as the trustee. In the middle of the TG, there was a programmed defection from the trustee. Thus the game not only examined trust formation but “dissolution” and “restoration” of trust as well. Their research found that those with high levels of BPD traits had difficulties forming trust in the beginning. Contrary to previous findings by King-Casas et al. (2008), the high-BPD group recognized the defection, but they increased transferred amounts at a markedly higher rate than the other groups after the defection. Surprisingly, this rate would attenuate after the restoration of cooperation by the trustee. Abramov et al. (2020) concluded that the high-BPD group’s trusting behavior is paradoxical: in response to continuous cooperation, their trust diminishes, to defection they respond much more generously than the low-BPD group. The paper also emphasized the characteristics of their study that could account for the differences between their findings and previous conclusions. First, their subjects played the role of the investor, and in this more dominant position, subjects might feel less need for aggressive retaliation because they are less defenseless, and second, the defection in their game was less ambiguous than that in the study by King-Casas et al. (2008).

Contrary to previous studies that showed a lack of cooperation in trust games in BPD, some studies concluded that fairness and active cooperation are characteristic features of BPD. A recent study by Lis et al. (2018) shows that BPD features are associated with higher justice sensitivity from a victim’s and an observer’s viewpoint, as well. Thielmann et al. (2014) show that higher levels of BPD features are associated with lower levels of HEXACO’s agreeableness and a tendency to retaliate in an ultimatum game. However, they found no such associations with the honesty-humility scale and active cooperation in a dictator game. The paper concluded that although individuals with higher levels of BPD features have difficulties with forgiveness and tolerance, they have a tendency to be fair, and therefore the paper ruled out to label BPD behavior as entirely non-cooperative. A study by Hepp et al. (2014) also examined the connection between BPD features and HEXACO. They found a negative association between BPD features and HEXACO’s agreeableness, a scale that incorporates reactive cooperation, forgiveness, and tolerance. However, they found no such associations with the honesty-humility scale of HEXACO which implies that BPD features do not influence fairness, i.e., active cooperation. Based on these findings, the question emerges: what is the basic social motivation of people with BPD? Do they strive to cooperate, and if they do what inhibits them from doing so? Our goal was to examine this question by utilizing the concept of social value orientation (SVO) and a simple method to measure it.

People’s basic disposition to what extent they are inclined to cooperate in interpersonal situations is described as their SVO. Decomposed games are simple tasks that measure SVO. These tasks do not enforce strategic thinking, only ask subjects to distribute specific amounts of money between themselves and another person as they prefer (Messick and McClintock, 1968). Based on one’s distribution, an SVO is attributed to the subject: prosocial, that can be further categorized into altruistic (1) or prosocial (2) types; or proself orientation, that can be further categorized into individualistic (3) or competitive (4) types (Bogaert et al., 2008). Amongst decomposed games, the most recent one was developed by Murphy et al. (2011), the Slider Measure of SVO. A notable feature of the Slider Measure is that it provides continuous SVO measurement, allowing us to measure primary social motivations more subtly (Murphy et al., 2011). As yet, no study examined SVO in BPD.

Actual cooperating behavior results from both the subject’s primary social motivation and her/his expectation (Pruitt and Kimmel, 1977; Pletzer et al., 2018). We presume that impaired cooperation in BPD is not due to their reluctance to cooperate but rather to their expectation of selfishness and disregard from others. Thus, our study’s goal was to examine BPD patient’s SVO and investigate their basic expectations about other people’s social motives. To do this, we used the Slider Measure –“Self-to-Other SVO”–, and we also modified the task to examine expectations –“Other-to-Self SVO.” In the Self-to-Other condition, subjects are asked to divide specific amounts of money between themselves and an unknown fictive other. In the Other-to-Self condition subjects have to divide the sums of money as they think the unknown fictive other would do it. We would like to capture the generalized representation of other people’s motives in our participants’ eyes with this task. We compared the results of the BPD group to a group of healthy volunteers. We presume that the vagueness of the second task (assuming the actions of an unknown fictive other) would activate relevant insecure internal working models or certain maladaptive schemas in the BPD group. Internal working models of attachment are known to be insecure attachment styles in BPD, such as unresolved or fearful type. These attachment styles include a longing for meaningful relationships that is restrained by mistrust and fear of rejection (Agrawal et al., 2004). Also self-to-other and other-to-self emotion schemes, that are developed in the infant and serve as tools to predict others’ reaction to the subject’s emotional needs could be of relevance when reporting expectations about the other’s fairness (Gergely and Unoka, 2008). The object relation dyads describe the internal representations of the self and others. In BPD these representations are usually split into all good/ideal or all bad/malevolent images (Levy et al., 2006). In addition, maladaptive schemas in the BPD group, such as *mistrust and abuse* (the person thinks that the other would deliberately exploit her/him) or *social isolation* (the person thinks that he/she is different from others, and does not belong to any group) schemas could also be relevant when reporting their expectations about the other’s SVO (Barazandeh et al., 2016).

Our main hypothesis was that there would be a significant difference between the two groups with respect to the Other-to-Self condition. Specifically, we presumed that BPD patients would expect a more selfish, individualistic, or competitive orientation, and therefore a larger difference between their own SVO and the other’s SVO. We based this hypothesis on the literature that indicates marked mistrust and negativity bias in BPD (Giesen-Bloo and Arntz, 2005; Unoka et al., 2011; Fertuck et al., 2013; Nicol et al., 2013; Roepke et al., 2013; Kleindienst et al., 2019). Our second hypothesis was that the two groups would not differ significantly regarding the Self-to-Other SVO. We base this hypothesis on findings that say that the majority of the healthy subject have prosocial orientation (Bogaert et al., 2008) and on previous literature that supports the notion that BPD patients value fairness and justice in social interactions (Hepp et al., 2014; Thielmann et al., 2014; Lis et al., 2018).

## Materials and Methods

### Participants

The patient group consisted of 30 subjects with BPD who participated in a 4-week psychotherapy program in the Department of Psychiatry and Psychotherapy at Semmelweis University, Budapest. Their diagnosis was established using the Structured Clinical Interview for DSM-5 Personality Disorders (First et al., 2016). Six patients were men (20%), mean age in the patient group was 26.27 years (*SD* = 6.74). As we did not collect information about the first appearance of the symptomatology in the patients’ lives, we can only give an estimate about the mean duration of the disorder. Since personality disorders begin in early adulthood or during the adolescent years (American Psychiatric Association, 2013), we calculated the mean duration of the disorder by subtracting 18 from the age at the time of assessment. Thus, the mean duration of the disorder was 8.27 years in the patient group.

The control group was a convenience sample of 30 healthy participants. Participants of the control group were recruited from the acquaintanceship of the staff with the intention to match the control sample to the BPD sample in terms of age, sex and education. Borderline PD symptoms of the control group were assessed with the Structured Clinical Interview for DSM-IV Personality Disorders Screen Questionnaire (First et al., 1997; Szádóczky et al., 2004). Participants were only included if their positive answers did not exceed two out of the 15 questions concerning BPD in the Screen Questionnaire. Ten subjects were men (33.3%), mean age in the control group was 25.7 years (*SD* = 6.85).

Comparison of demographic data and clinical description of the patient group are shown in Table 1.

**TABLE 1:** Demographics and clinical description of the BPD group.

	Group					
	
	BPD		CTRL		Statistical test	
	(*N* = 30)		(*N* = 30)				
				
	Mean	*SD*	Mean	*SD*	*t*(58)	*p*
Age (years)	26.27	6.74	25.7	6.85	−0.323	0.748
	N	%	N	%	χ^2^	
Education					7.407	0.192
Primary school	5	16.67	1	3.33		
Vocational school	3	10	1	3.33		
Vocational school with high school diploma	5	16.67	2	6.67		
Grammar school	12	40	21	70		
College	3	10	3	10		
University	2	6.67	2	6.67		
Gender					1.364	0.243
Female	24	80	20	66.67		
Male	6	20	10	33.33		
Medication	N	%				
Antidepressants	19	63.33				
Benzodiazepines	16	53.33				
Mood stabilizers	9	30				
Antipsychotics (atypical only)	15	50				
Co-occurring mental disorders						
Obsessive-compulsive personality disorder	7	23.33				
Avoidant personality disorder	6	20.00				
Paranoid personality disorder	5	16.67				
Dependent personality disorder	2	6.67				
Narcissistic personality disorder	1	3.33				
Histrionic personality disorder	1	3.33				
Bipolar disorder	11	36.67				
Depression	10	33.33				
Eating disorder	6	20.00				
Panic disorder	4	13.33				
Anxiety disorder	2	6.67				
PTSD	2	6.67				
Adjustment disorder	1	3.33				
ADHD	1	3.33				
Somatoform disorder	1	3.33				

*BPD, borderline personality disorder; CTRL, control group; PTSD, post-traumatic stress disorder; ADHD, attention deficit hyperactivity disorder.*

Semmelweis University’s Regional, Institutional Scientific and Research Ethics Committee gave consent to conduct the study.

### Procedure

Participants provided informed consent prior to the administration of the test. The test was taken individually in the presence of the examiner in a paper-based format. The administration of the test took about 15 min. Participants of the patient group were tested during the first week of the 4-week psychotherapy program.

### Measures

Slider Measure of Social Value Orientation –Self-to-Other condition. The Slider Measure was developed by Murphy et al. (2011). In the task, participants have to make 15 decisions about dividing sums of money between themselves and a fictive unknown person. Each of the 15 items of the task consists of 9 possible divisions. The participants have to choose one of the 9 possibilities. For example, it contains the following possible choices: 50 (you receive)-100 (other receives), 54 (you receive)-98, (other receives), 59 (you receive)-96 (other receives), 63 (you receive), 94(other receives), 68 (you receive)-93 (other receives), 72 (you receive), 91(other receives), 76 (you receive)-89 (other receives), 81 (you receive), 87 (other receives), 85 (you receive)-85 (other receives). The task provides a categorization of subjects into four SVOs: altruist, prosocial, individualist, and competitive. The test also provides a continuous scale of SVO, the SVO-angle: if it is less than −12.04° it shows competitive orientation. If it is between −12.04° and 22.45° it shows individualistic orientation. If it is between 22.45° and 57.15° it shows prosocial orientation, and if it is more than 57.15°, it shows altruistic orientation. The psychometric properties of the Slider Measure are satisfactory (Murphy et al., 2011). Its author granted permission to use the task in our study. The task and scoring are available at the author’s website.^1^

SVO Slider Measure—Other-to-Self condition. This task is the modification of the original Slider Measure that we created to examine subjects’ expectations from other people. Instruction of the task was: “In the following task, imagine that the other person from the previous task was given the same instructions as you. What do you presume his/her answers would be?” We also switched the labels “you receive” and “other receives.” The allocation decisions were the same as in the original task. The task provides the Other-to-Self SVO angle a continuous variable to describe people’s expectations.

We computed our main variable by subtracting the Other-to-Self SVO angle from subjects’ own SVO angle, the SVO angle difference. We created this variable to capture the difference between subjects’ own SVO and their expectations from other people. Positive values of this variable indicate that a prosocial self expects proself orientation from the other. Negative values indicate that a proself subject expects more prosocial orientation than his/her own SVO.

### Statistical Analysis

We used IBM SPSS Statistics 25 for data analysis. We conducted Shapiro-Wilk tests to check if our data is normally distributed. Our main variable did not meet this expectation. Therefore, we used Man-Whitney *U*-test for its analysis. Other continuous variables were compared in Independent-Samples *T*-tests.

## Results

### Comparison of SVO Angle Differences

**FIGURE 1:**
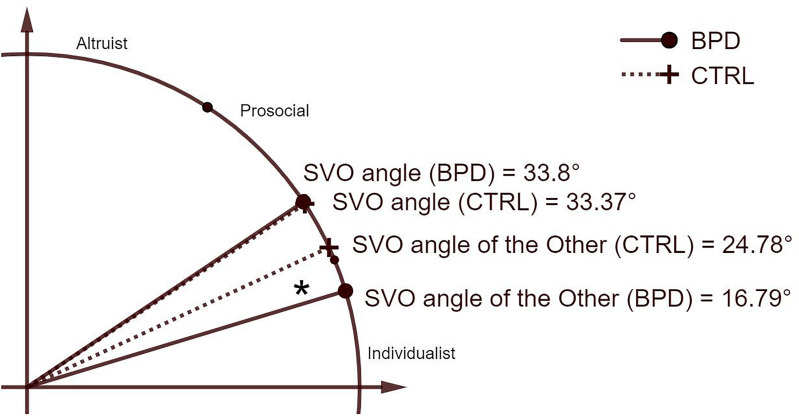
SVO angle differences in the BPD and in the control group. Differences between the borderline (BPD) group and the control (CTRL) group regarding continuous variables of the tasks are depicted in the top right quadrant in the Cartesian plane. Boundaries between SVO categories are indicated with smaller dots (SVO, social value orientation).

Since Shapiro-Wilk test showed that data in the control group was not normally distributed, W(30) = 0.823; *p* < 0.001, we applied Mann-Whitney U test to compare group differences. Results of the test showed that the SVO angle differences were significantly greater in the BPD (Mdn = 36.53) than in the control group (Mdn = 24.47), *U* = 269, *z* = −2.684, *p* = 0.007, *r* = 0.346 (Figure 1). This result indicates that the BPD group expects significantly more proself orientation relative to their own SVO than the control group.

### Other-to-Self SVO Angle

Comparing the Other-to-Self SVO, we found significant differences *t*(58) = 2.211, *p* = 0.031, between the BPD (*M* = 16.79, *SD* = 15.24) and the control group (*M* = 24.78, *SD* = 12.63). Since a larger angle indicates less selfish, prosocial behavior, this result also shows that the BPD group expects the other to be significantly more selfish and individualistic than the control group.

### Self-to-Other SVO Angle

Comparing the SVO angle of the BPD (*M* = 33.8, *SD* = 13.48) and control group (*M* = 33.37, *SD* = 8.67), we found no significant differences: *t*(58) = −0.145, *p* = 0.885.

## Discussion

The aim of this study was to examine SVO in a group of BPD patients and to assess their expectations regarding other people’s SVO.

In harmony with our main hypothesis we found significant differences between the two groups regarding the expectations about the other’s SVO. The BPD group expected significantly more proself orientation from the other, while their own SVO did not differ significantly from that of the control group. Comparison of the difference between subjects’ own SVO and their expectation from the other yielded significant results with medium effect size. This difference was significantly greater in the BPD group, indicating that the prosocial patients expect more individualistic, selfish attitudes from others than the prosocial controls; that is, patients see bigger differences between their own and the other’s prosocial motivations. This is in line with previous literature reporting of the diverse examples of negativity bias in BPD (Roepke et al., 2013), specifically with that of Giesen-Bloo and Arntz (2005), who examined world assumptions in BPD and found that BPD patients see the world and others significantly more malevolent than the comparison groups (a group of healthy controls, a group with cluster C PDs and a group with Axis I pathology). Our results are also in accordance with the conclusions of King-Casas et al. (2008), who say that proper reaction to defection by a partner in a trust game and the ability to restore the cooperation is impaired in BPD because these patients fail to detect defection since it does not fall short from their own expectations. In line with this explanation, Polgár et al. (2014) found that BPD patients accept unfair offerings in an ultimatum game significantly more often than the control group. In the study of Unoka et al. (2009), in a series of trust games, BPD participants invested significantly less than controls and did not increase their investments during the task as opposed to controls. An essential feature of the study was that they did not inform the subjects about back-transfers from the partner to let them rely solely on their mental representation about their interaction partner’s potential SVO during their investment decisions. Similarly, in our second task, subjects are instructed to imagine an unknown other who is in the position to decide about their “fortune.” This is also an uncertain situation where they respond similarly as in the study of Unoka et al. (2009): with a lack of trust and the presumption of small transfers from a stranger. Our findings also support the conclusion that individuals with high levels of BPD features exhibit paradoxical trusting behaviors examined in a 15-round trust game (Abramov et al., 2020). Abramov et al. (2020) found that a partner’s cooperative behavior is paired with mistrust from the high-BPD individual (decreasing offers) while defection by the partner is paired with an early increase in generous offers. The partner’s defection in the trust game coincides better with the internal representation of other people’s motives in BPD that is shown in our results: BPD patients expect selfishness despite their own prosocial behavior. Thus, it is possible that the initial cooperation of a partner in the trust game is unfamiliar, confusing, and a cause for caution in BPD, and results in mistrustful behavior.

Our secondary hypothesis was that patients’ own SVO would not differ significantly from the control group’s SVO. In harmony with our hypothesis, we did not find significant differences when comparing the SVO of the two groups. It has been established that people with BPD are sensitive to injustice (Lis et al., 2018) and that even though actual cooperative behavior is impaired in BPD (King-Casas et al., 2008; Unoka et al., 2009), most likely the reactive part of cooperation—that is the ability to forgive and not retaliate -that shows impairment not their proneness to be fair (Hepp et al., 2014; Thielmann et al., 2014).

Considering that early maltreatment, neglect and abuse is an important etiological factor in BPD (Zanarini et al., 1997), this pattern of social motivation might derive from a family environment where cooperation of the child was obligatory whereas it was not reciprocated by the environment, rather the cooperation met with selfishness and disregard for the needs of the child.

## Limitations

First of all, the sample sizes are relatively small. Second, in the absence of a patient control group we cannot state that our findings are specific to BPD and not the result of general psychopathology. Also, comorbidities in the patient group could have influenced our results. Another limitation is that we examined the dispositions of the subjects in hypothetical situations. We did not assess actual cooperative behavior in an interaction with a partner. Thus, we can only assume the connection between our findings and the interpersonal problems in BPD. On the other hand, the absence of a partner also made it possible to examine our participants’ expectations in ambiguous circumstances. Moreover, our study could have benefited from including an assessment of a specific feature that is characteristic of BPD, in order to better understand how our findings fit into the BPD symptomatology. For example negative affect or intolerance of uncertainty are both characteristic features of BPD and could contribute to our findings. Recent study has showed that intolerance of uncertainty contributes to negative affect possibly through enhancing the need for maladaptive emotion regulation strategies, thus it could be an important factor in patients’ lives when encountering stressful and ambiguous situations (Bottesi et al., 2018). In our study we examined the subjects’ social motivations and expectations in an ambiguous situation where their fictive partner was a completely unknown person, thus, they needed to fill this gap of uncertainty from their own past experiences. It is possible that such a situation, even though hypothetical, in itself creates frustration in patients with BPD. That frustration could also shape their answers further on, consequently adding to the negative bias when reporting their expectations about the partner’s social motives. Also, this negative bias could be associated with heightened negative affect in individuals with BPD. Finally, there is a growing need for a refined definition of BPD and the solution most likely resides in replacing our traditional categorical mind-set regarding personality disorders with a more dimensional approach (Sharp, 2018). The need for a dimensional approach has been recognized and specifically addressed by both the 5th edition of the Diagnostic and Statistical Manual of Mental Disorders (DSM-5) and the 11th edition of the International Classification of Diseases (ICD-11), and there is a growing literature that examines the measures and concepts of these models, thus facilitating the utilization and amelioration of these dimensional approaches (Bach and First, 2018; Zimmermann et al., 2019). Although, it was not an aim of this study to address this question, the application of a dimensional approach in our study would have increased the value of our findings.

## Conclusion

Social value orientation is about our basic social motivations, about our willingness to take into account other people’s interest when making decisions. To our knowledge SVO has not been investigated in BPD, so far. Our findings indicate that BPD patients are ready to consider other people’s interest when making decisions just as much as healthy subjects, i.e., their interpersonal problems cannot be explained by a lack of prosocial disposition. However, their basic expectations of selfish behavior from other people can contribute both to problems in cooperation and everyday social difficulties. This internal representation could play an important role in everyday ambiguous situations when reassurance of benevolence or explicit indication of an intention to cooperate is absent from another person. This absence may be automatically substituted with their basic expectation of selfishness.

## Data Availability Statement

The original contributions presented in the study are included in the article/supplementary material, further inquiries can be directed to the corresponding author/s.

## Ethics Statement

The studies involving human participants were reviewed and approved by the Semmelweis University’s Regional, Institutional Scientific and Research Ethics Committee. The patients/participants provided their written informed consent to participate in this study.

## Author Contributions

EL took part in data collection and analysis, conceptualization, methodology and the writing process, prepared the original draft and further on reviewed, and edited the article. BB took part in data collection and the writing process, reviewed, and edited the article. ZU took part in conceptualization, methodology and the writing process, reviewed, and edited the article. All authors contributed to the article and approved the submitted version.

## Conflict of Interest

The authors declare that the research was conducted in the absence of any commercial or financial relationships that could be construed as a potential conflict of interest.

## Publisher’s Note

All claims expressed in this article are solely those of the authors and do not necessarily represent those of their affiliated organizations, or those of the publisher, the editors and the reviewers. Any product that may be evaluated in this article, or claim that may be made by its manufacturer, is not guaranteed or endorsed by the publisher.

## Footnotes

1
http://ryanomurphy.com/styled-2/index.html
